# N-acetylcysteine nano-spray versus conventional treatment in the management of radiotherapy-induced oral mucositis in oral cancer patients: a randomized clinical trial

**DOI:** 10.1186/s12903-026-07959-7

**Published:** 2026-03-20

**Authors:** Alaa Essam, Shahira El Domiaty, Nahed Attia, Hala Helal, Mohamed Meheissen

**Affiliations:** 1 Oral Medicine, Periodontology, Oral Diagnosis, and Oral Radiology Department, Faculty of Dentistry, Alexandria University, Alexandria, Egypt; 2 Industrial Pharmacy Department, Faculty of Pharmacy, Alexandria University, Alexandria, Egypt; 3 Clinical Oncology and Nuclear Medicine Department, Faculty of Medicine, Alexandria University, Alexandria, Egypt

**Keywords:** Radiotherapy, Oral mucositis, N-acetylcysteine, Nano-spray, WHO, OHIP-14, Gastrin-17

## Abstract

**Background:**

Radiation-induced oral mucositis (RIOM) is a common and debilitating complication of radiotherapy in oral cancer patients, significantly impairing quality of life and potentially interrupting treatment. This study evaluated the therapeutic efficacy of N-acetylcysteine (NAC) nano-spray in managing RIOM, with particular emphasis on mucositis severity, quality of life, and serum gastrin-17 levels.

**Materials and methods:**

In this randomized clinical study, 40 oral cancer patients undergoing radiotherapy were allocated in a 1:1 ratio to receive either NAC nano-spray (*n* = 20) or conventional therapy (*n* = 20) for six weeks. Mucositis severity was assessed using the World Health Organization (WHO) Oral Mucositis Severity Scale, and quality of life was evaluated using the Oral Health Impact Profile (OHIP-14). Serum gastrin-17 levels were measured before and after treatment.

**Results:**

Patients treated with NAC nano-spray demonstrated a significant reduction in WHO mucositis grades and significantly improved OHIP-14 scores compared with the control group (*p* < 0.05). Additionally, a significant increase in serum gastrin-17 levels was observed in the NAC group relative to conventional therapy (*p* < 0.05). These effects were evident during radiotherapy and at the end of treatment.

**Conclusions:**

N-acetylcysteine nano-spray appears to be an effective therapeutic option for the management of RIOM, reducing mucositis severity and improving quality of life in oral cancer patients.

**Trial registration:**

The trial was registered at Clinical Trials .gov on 24/7/2025, registration number: (NCT07082621).

**Supplementary Information:**

The online version contains supplementary material available at 10.1186/s12903-026-07959-7.

## Background

Radiation-induced oral mucositis (RIOM) is a common and debilitating side effect of radiotherapy and chemotherapy, progressing through five stages: initiation, primary damage response, signal amplification, ulceration, and healing.DNA damage in basal epithelial cells triggers reactive oxygen species (ROS) formation, leading to inflammation, epithelial thinning, and mucosal injury. Ulcers formed during the ulceration stage are often colonized by bacteria, and healing generally begins after radiotherapy ends [1]. RIOM causes severe pain and discomfort, often necessitating hospitalization for hydration, nutrition, pain management, and infection control, which increases healthcare costs [2].

Effective management of RIOM is essential to improve quality of life. Preventive oral care including hygiene instruction, fluoridated toothpaste, antiseptic mouthwash, calculus removal, and avoidance of alcohol and spicy foods reduces mucositis severity, alleviates pain, and decreases reliance on systemic analgesics [3]. Symptomatic treatment options include topical anesthetics, mucosal coating agents, antibiotics, antifungals, steroids [4], and non-steroidal anti-inflammatory agents such as benzydamine mouthwash [5]. Certain vitamins and herbal supplements act as free radical scavengers to mitigate oxidative mucosal damage [6]. Low-level laser therapy (LLLT) or photobiomodulation therapy (PBMT) promotes tissue repair and reduces inflammation through photophysical and biochemical modulation [7], while cryotherapy has also shown favorable outcomes [8].

N-acetylcysteine (NAC), a glutathione precursor and potent antioxidant, reduces inflammatory cytokine production and inhibits NF-ΚB activation, helping to control tissue damage associated with RIOM [9–12]. NAC has been shown to protect against radiation-induced side effects and reduce the incidence and severity of RIOM when administered systemically or as an oral rinse [13–16]. Chitosan, a biodegradable, biocompatible, and mucoadhesive polymer, enhances the stability, bioavailability, and mucosal delivery of NAC nanoparticles [17–19].

Serum Gastrin-17 (G-17) is a sensitive biomarker of gastric mucosal structure and function. RIOM-related pain and reduced oral intake can decrease G-17 levels, which may normalize following effective treatment. Baseline G-17 measurement may help identify patients at higher risk for oral mucositis and guide personalized nutritional interventions [20–25].

To our knowledge, no studies have evaluated NAC-based nanoparticles for RIOM in oral cancer patients. This study aimed to assess the clinical effectiveness of NAC-loaded nanoparticles in reducing RIOM severity and to evaluate changes in serum G-17 levels as a potential biomarker of nutritional and mucosal status during radiotherapy.

## Materials and methods

### Study design

A randomized clinical trial was conducted on 40 patients diagnosed with oral cancer, in accordance with the ethical principles outlined in the Declaration of Helsinki and following the CONSORT reporting guidelines for reporting randomized controlled trials [26, 27]. Participants Patients were recruited from the outpatient clinics at Alexandria University and Ayady Elmostakbal Oncology Hospital.

The study was approved by received ethical approval from the Institutional Review Board (IRB No.) under the approval number IRB NO 00010556 – IORG0008839–0727-07/2023) and was officially registered on ClinicalTrials.gov (NCT07082621) on July 24, 2025. The full, with the registration number NCT07082621. A detailed trial protocol is available and can be provided upon request.

### Participants

Forty adult patients (≥ 20 years of age) of both sexes with oral cancer undergoing radiotherapy (RT) at recruitment were enrolled. Radiotherapy was administered using the Elekta Unity linear accelerator as either postoperative or definitive treatment [28]. All participants clinically diagnosed radiotherapy-induced oral mucositis (RIOM) with varying severity at enrollment [29]. Patients with other malignancies, systemic diseases, anticoagulant use, or physical or mental impairments were excluded [30].

### Sample size estimation

Sample size estimation was performed using a 5% alpha error and 80% power, based on the method described by Tsujimoto et al. [31]. In that study, the mean ± SD mucositis grade was 2.9 ± 0.3 in the glutamine group and 3.3 ± 0.4 in the control group. The highest standard deviation (0.4) was applied, yielding a required sample of 17 patients per group. To compensate for potential attrition, the sample size was increased to 19–20 patients per group, for a total of 40 participants. Calculations followed Rosner’s method [32] and were performed using G*Power version 3.1.9.7 [33].

### Randomization, blinding, and allocation concealment

Participants were randomized using a computer-generated sequence with equal allocation, and baseline demographic and clinical characteristics were compared between groups to confirm randomization adequacy, in accordance with CONSORT guidelines [27, 34].

Blinding of participants and the primary operator was not feasible due to the nature of the interventions. The control group received multiple therapeutic agents, including topical analgesics and antifungal/anti-inflammatory mouthwashes with variable dosing regimens, whereas the test group received a NAC nano-spray only. Development of a matching placebo was impractical and ethically unacceptable, as withholding active treatment would have subjected patients with severe oral mucositis to significant pain.

To minimize bias, outcome assessment was blinded. Clinical evaluations were performed by a blinded oral medicine specialist, classifying the trial as a randomized open blinded endpoint (PROBE) design, as defined by Hansson et al. [35].

Oral mucositis was assessed using the World Health Organization (WHO) Oral Mucositis Grading Scale following examiner training with standardized clinical images representing all grades (0–4). Intra-examiner calibration was performed through repeated evaluations of a pilot set of images at two times, yielding a Kappa coefficient of 0.82 [36], indicating excellent reliability and consistent mucositis scoring throughout the study.

### Preparation of NAC-chitosan nanoparticles spray [37]

Chitosan, sodium tripolyphosphate, and N-acetylcysteine (NAC) (Sigma-Aldrich) were used to synthesize chitosan–NAC (CS-NAC) nanoparticles using the ionic gelation method, with sodium tripolyphosphate as the crosslinking agent. Chitosan was dissolved in 1% acetic acid and added to the CS-NAC solution under continuous stirring for 1 h. NAC concentration was quantified using high-performance liquid chromatography (HPLC). The resulting nanoparticles were freeze-dried and stored at 4 °C. 

#### Culture conditions and cell lines

The Stem Cell Technology Research Center provided human colon cancer cell lines, cultured in DMEM with fetal bovine serum, and maintained at 37 °C with 5% CO2.

#### Assay for cytotoxicity

The cytotoxicity of NAC and NAC-chitosan nanoparticles on a colon cancer cell line was tested using the MTT assay. Significant differences were identified using SPSS software, expressing the impact on cell viability [38].

#### Physicochemical characterization of NAC-chitosan nanoparticles

Using a dynamic light scattering (DLS) method and a NanoZS/ZEN3600 Zetasizer (Malvern Instruments, Malvern, UK), the mean particle size (Z-average size), polydispersity index (PDI), and zeta potential (ZP) were determined.

This system uses a 4-mW helium/neon laser with a wavelength of 633 nm to measure particle size at a detection angle of 173° using noninvasive backscattering technology. To prevent the multi-dispersion of light brought on by a high concentration, NAC-chitosan nanoparticles dispersions were diluted with filtered water (1:20) before measuring. All the measurements were performed in triplicate.

### Intervention

Forty patients diagnosed with radiation-induced oral mucositis (RIOM) were randomly allocated into two groups. The control group (*n* = 20) received conventional therapy for six weeks, consisting of topical anesthetic^1^ (1.5 g benzocaine oral spray), topical analgesic^2^ (2.0 g lidocaine hydrochloride gel), topical antifungal^3^ (miconazole 2% oral gel administered twice daily using a standardized spoon), and anti-inflammatory mouthwash^4^ (5 g sodium bicarbonate solution used twice daily with a standardized measuring cup). Systemic analgesics were administered as required for pain control [39].

Twenty patients received N-acetylcysteine (NAC) nano-spray at a total daily dose of 1800 mg. The formulation was administered three times daily, with 10 sprays every eight hours using a long-nozzle device designed to access otherwise inaccessible oral areas [29]. This administration protocol was developed in collaboration with the Industrial Pharmacy Department, Faculty of Pharmacy, Alexandria University.

Systemic analgesics were provided as needed for pain control. Prophylactic antifungal therapy was not routinely administered; however, patients were clinically monitored throughout the study period for signs of oral candidiasis. Rescue antifungal therapy (topical miconazole or systemic fluconazole) was initiated when clinically indicated.

All patients with radiation-induced oral mucositis (RIOM) were managed in accordance with the MASCC/ISOO clinical practice guidelines for basic oral care. Comprehensive medical and dental histories were obtained, followed by detailed intraoral examinations. Standardized oral hygiene instructions were provided, including the use of fluoridated toothpaste, adequate hydration, and smoking cessation counseling [40].

All participants received a brief explanation of the study objectives and procedures and provided written informed consent detailing potential outcomes, adverse effects, and the right to withdraw at any time. To enhance adherence, participants were provided with self-monitoring reminder sheets outlining dosage instructions.

Patients presenting with grade 3–4 oral mucositis were re-evaluated by oncology specialists, and additional interventions—including parenteral nutrition, systemic analgesics, systemic antifungals, or hospitalization—were implemented when clinically indicated [39].

### Outcome measures

Radiation-induced oral mucositis (RIOM) typically developed during the second or third week of radiotherapy. Patients were clinically evaluated throughout the treatment course and at its completion. Objective assessment was performed at baseline and at six weeks using the World Health Organization (WHO) Oral Mucositis Grading Scale to determine RIOM severity, based on functional impairment and clinical signs and symptoms [41], as outlined in Table 1.

Subjective assessment was performed using the Oral Health Impact Profile (OHIP), a validated self-reported questionnaire that evaluates the impact of oral health conditions on quality of life and daily functioning. The original OHIP-49 consists of 49 items across seven domain functional limitations, physical pain, psychological discomfort, physical disability, psychological disability, social disability, and handicapped based on Locker’s conceptual model of oral health.

The shortened version, OHIP-14, retains the same seven domains using two representative items per domain, with responses scored on a 5-point Likert scale (0–4). The total score ranges from 0 to 56, with higher scores indicating poorer oral health–related quality of life. OHIP-14 was used to assess changes in subjective symptoms and quality of life following N-acetylcysteine (NAC) application at 2, 4, and 6 weeks of follow-up [42], as shown in Table 2.

**Table 1:** WHO grading system for Oral mucositis severity

Grade	Description
Grade 0	None
Grade I	Mild mucositis: oral soreness and/or erythema
Grade II	moderate mucositis: oral erythema, ulcers, and a solid diet can be tolerated
Grade III	Severe mucositis: oral ulcers, but a solid diet cannot be tolerated
Grade IV	Life-threatening: oral alimentation is impossible

**Table 2:** Oral Health Impact Profile 14 (OHIP-14): -

1. Have you had trouble pronouncing any words because of problems with your teeth, mouth, or dentures?
2. Have you felt that your sense of taste has worsened because of problems with your teeth, mouth, or dentures?
3. Have you had painful aching in your mouth?
4. Have you found it uncomfortable to eat any foods because of problems with your teeth, mouth, or dentures?
5. Have you been self-conscious because of your teeth, and mouth or dentures?
6. Have you felt tense because of problems with your teeth, mouth, or dentures?
7. Has your diet been unsatisfactory because of problems with your teeth, mouth, or dentures?
8. Have you had to interrupt meals because of problems with your teeth, mouth, or dentures?
9. Have you found it difficult to relax because of problems with your teeth, mouth, or dentures?
10. Have you been a bit embarrassed because of problems with your teeth, mouth, or dentures?
11. Have you been a bit irritable with other people because of problems with your teeth, mouth, or dentures?
12. Have you had difficulty doing your usual jobs because of problems with your teeth, mouth, or dentures?
13. Have you felt that life, in general, was less satisfying because of problems with your teeth, mouth, or dentures?
14. Have you been unable to function because of problems with your teeth, mouth, or dentures?

### Biochemical evaluation

#### Sample collection and storage

Participants fasted for more than 10 h prior to blood sample collection. Blood samples were centrifuged to separate serum, which was stored at − 20 °C–− 80 °C until analysis.

Serum gastrin-17 levels were measured at baseline and again after six weeks of radiation-induced oral mucositis (RIOM) treatment [25, 43]. Measurements were performed using the Human Gastrin ELISA Kit (Catalog No. DL-GT-Hu, 96 tests) purchased from DIDevelop Multinational Company. All biochemical analyses were conducted at the Genomics Research Laboratory, Medical Research Center, Faculty of Medicine, Alexandria University.

#### Statistical analysis

Data normality was assessed using the Shapiro–Wilk test, Q–Q plots, and descriptive statistics. Age, total OHIP-14 scores, and serum gastrin-17 levels were normally distributed, whereas oral mucositis severity scores were non-normally distributed. Normally distributed variables were expressed as mean ± standard deviation, and non-normally distributed variables as median [interquartile range]. Linear mixed-effects models were used to evaluate intervention × time effects, with group and time as fixed effects, participants as random intercepts (unstructured covariance matrix), and radiation dose and age as covariates. All tests were two-tailed, and p < 0.05 was considered statistically significant. Statistical analyses were performed using IBM SPSS Statistics for Windows, version 23.0 (IBM Corp., Armonk, NY, USA).

## Results

**Fig. 1:**
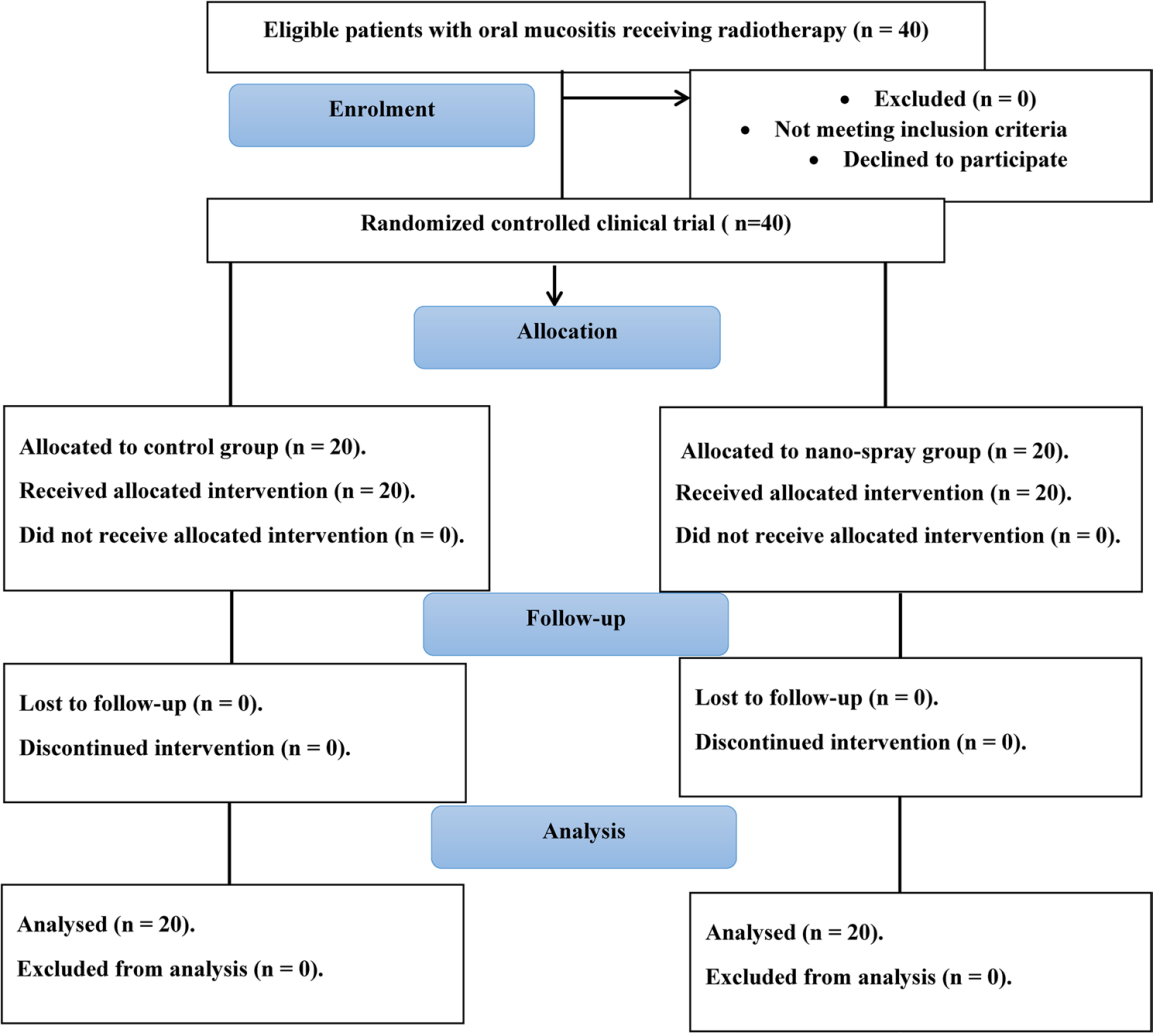
CONSORT flow diagram

A total of 40 oral cancer patients were assessed for eligibility (Fig. 1). The study group (NAC nano-spray) included 11 males (55%), while the control group included 8 males (40%). The mean age was 43.75 ± 13.17 years in the study group and 52.50 ± 7.91 years in the control group.

Baseline demographic and clinical characteristics of the two groups are presented in Table 3. The study group was significantly younger than the control group (43.75 ± 13.17 vs. 52.50 ± 7.91 years; *p* = 0.015) and received a higher mean radiation dose (*p* = 0.017). To account for these differences, a linear mixed-effects model was performed with age and radiation dose as covariates, confirming that the treatment effect remained significant independent of these baseline imbalances. Radiation dose and age did not show significant effects on outcomes (Supplemental Table 1). No significant differences were observed between groups regarding gender distribution, tumor site, or type of radiation therapy (*p* > 0.05), with the tongue being the most common tumor site in both groups.

Topical antifungal prophylaxis was not routinely administered in the NAC group. During the study, six patients in the NAC group required rescue antifungal therapy, compared with four patients in the control group, reflecting differences in antifungal management between groups.

**Fig. 2:**
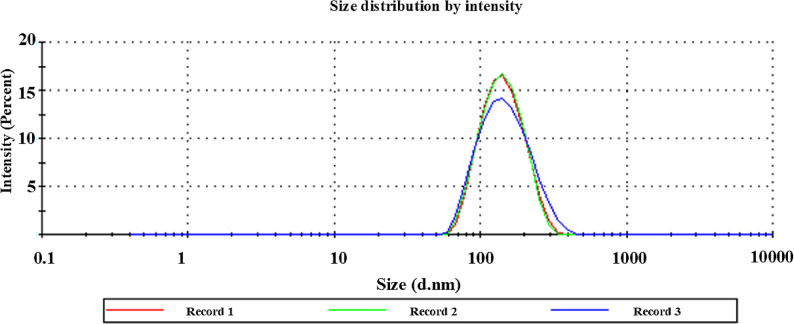
Particle size distribution diagram of NAC-chitosan nanoparticles

The NAC–chitosan nanoparticles had a mean particle size of 171.6 ± 3.06 nm and a polydispersity index (PDI) of 0.293, indicating a homogeneous size distribution (Fig. 2).

Regarding safety and tolerability, the most reported complaints associated with topical NAC nano-spray were a bitter taste and unpleasant oral odor. No other adverse effects were observed in either group throughout the study.

**Table 3:** Baseline characteristics

		Study Group (*n* = 20)	Control Group (*n* = 20)	*p* value
Age in years	Mean ± SD	43.75 ± 13.17	52.50 ± 7.91	0.015*
Gender: n (%)	Males	11 (55%)	8 (40%)	0.342
	Females	9 (45%)	12 (60%)	
Diagnosis: n (%)	Buccal mucosa	1 (5%)	0 (0%)	0.654
	Gingival	1 (5%)	0 (0%)	
	Lip	0 (0%)	1 (5%)	
	Mandible	3 (15%)	3 (15%)	
	Maxilla	3 (15%)	1 (5%)	
	Parotid Gland	1 (5%)	1 (5%)	
	Tongue	10 (50%)	11 (55%)	
	Tonsils	1 (5%)	3 (15%)	
Radiation therapy: n (%)	Adjuvant	8 (40%)	11 (55%)	0.342
	Definitive	12 (60%)	9 (45%)	
Radiation dose	Mean ± SD	67.00 ± 2.99	64.00 ± 4.47	0.017*

*Statistically significant difference at p value < 0.05

Oral mucositis severity improved significantly in the NAC nano-spray group over six weeks. At baseline, both groups had similar severity distributions (*p* = 0.173). By week six, 95% of the study group had grade 1 mucositis compared with 30% of controls (*p* = 0.003). Mean severity scores decreased from 2.70 ± 0.80 to 1.10 ± 0.45 in the study group (*p* < 0.001), whereas the control group showed a smaller, non-significant reduction (2.45 ± 1.05 to 2.20 ± 0.95; *p* = 0.155). These results indicate that NAC nano-spray substantially reduced mucositis severity compared with conventional care as shown in (Table 4).

**Table 4:** Comparison of WHO oral mucositis grade between the control and study group at different timepoints

Follow up	Scales		Study Group (*n* = 20)	Control Group (*n* = 20)	*p* value
Baseline	Severity levels: n (%)	Grade 1	1 (5%)	6 (30%)	0.173
		Grade 2	7 (35%)	1 (5%)	
		Grade 3	9 (45%)	11 (55%)	
		Grade 4	3 (15%)	2 (10%)	
	Severity scores	Mean ± SD	2.70 ± 0.80	2.45 ± 1.05	
		Median (IQR)	3.00 (1.00)	3.00 (2.00)	
6th week	Severity levels: n (%)	Grade 1	19 (95%)	6 (30%)	0.003*
		Grade 2	0 (0%)	5 (25%)	
		Grade 3	1 (5%)	8 (40%)	
		Grade 4	0 (0%)	1 (5%)	
	Severity scores	Mean ± SD	1.10 ± 0.45	2.20 ± 0.95	
		Median (IQR)	1.00 (0.00)	2.00 (2.00)	
*p* value^2^			< 0.001*	0.155	

* Statistically significant difference at p value<0.05

**Fig. 3:**
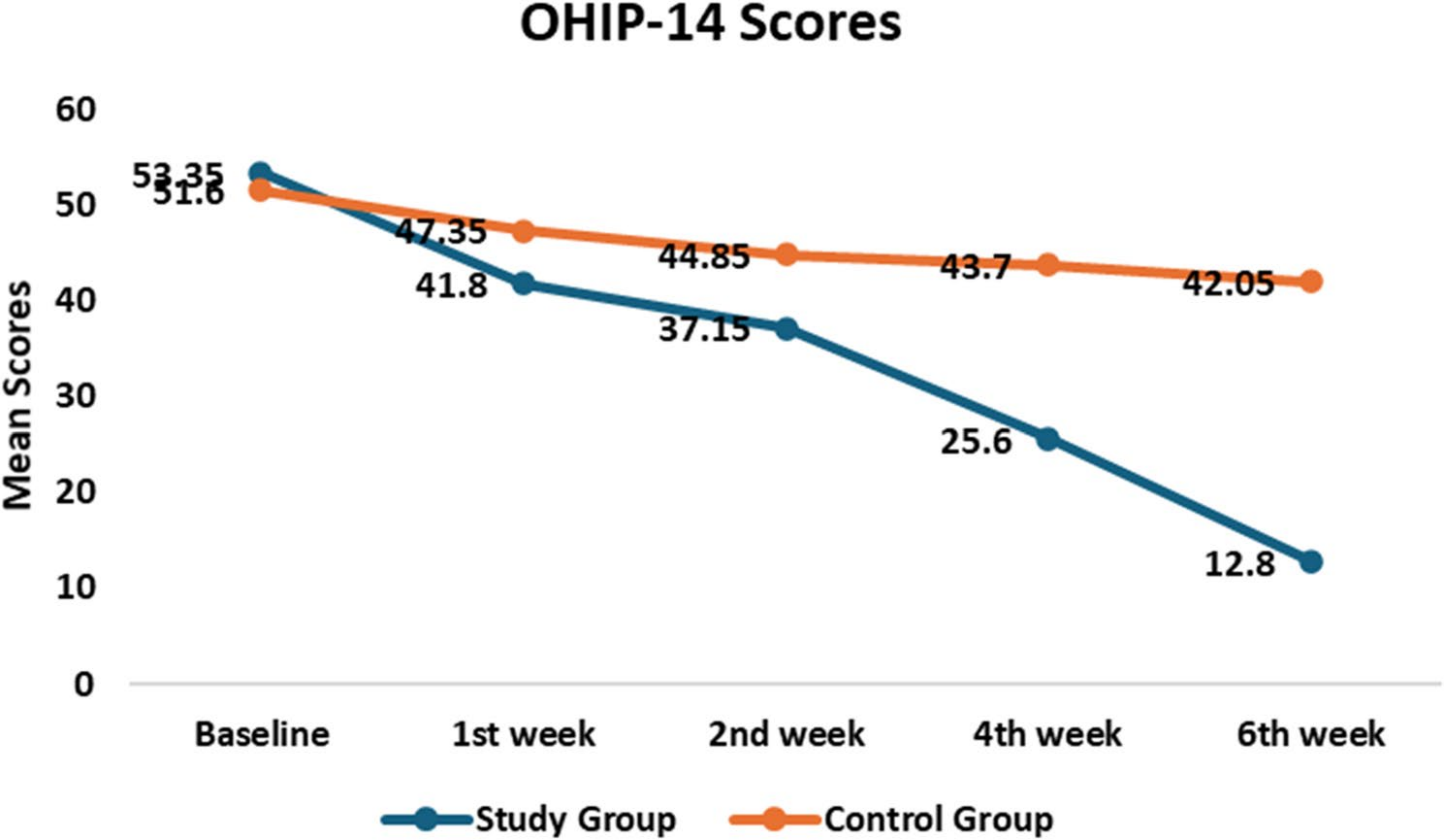
Comparison of OHIP-14 scores between the control and study groups at different timepoints

Figure 3 shows that the study group experienced a greater reduction in OHIP-14 scores over six weeks compared to the control group, reflecting significantly improved oral health–related quality of life (*p* < 0.001).

Oral health–related quality of life, assessed by OHIP-14, improved progressively over six weeks in both groups, but the effect was more pronounced in the NAC nano-spray group. In the study group, OHIP-14 scores decreased significantly at each follow-up compared with baseline (*p* < 0.001), with consistent improvements observed between consecutive time points. In contrast, the control group showed smaller, less consistent reductions; some intervals, such as week 2 to week 4 and week 4 to week 6, did not reach statistical significance (*p* > 0.05). Overall, these results indicate that NAC nano-spray accelerated recovery of oral health–related quality of life compared with conventional management as presented in Table 5 and Supplemental Table 2.

**Table 5:** Pairwise comparisons of OHIP-14 scores between follow-up intervals within each group

Time	Compared to	*p* value	
		Study Group	Control Group
Baseline	1st week	< 0.001*	0.003*
	2nd week	< 0.001*	< 0.001*
	4th week	< 0.001*	< 0.001*
	6th week	< 0.001*	< 0.001*
1st week	2nd week	0.001*	0.296
	4th week	< 0.001*	0.078
	6th week	< 0.001*	0.003*
2nd week	4th week	< 0.001*	1.00
	6th week	< 0.001*	0.406
4th week	6th week	< 0.001*	1.00

*Statistically significant difference at *p* value < 0.05

**Fig. 4:**
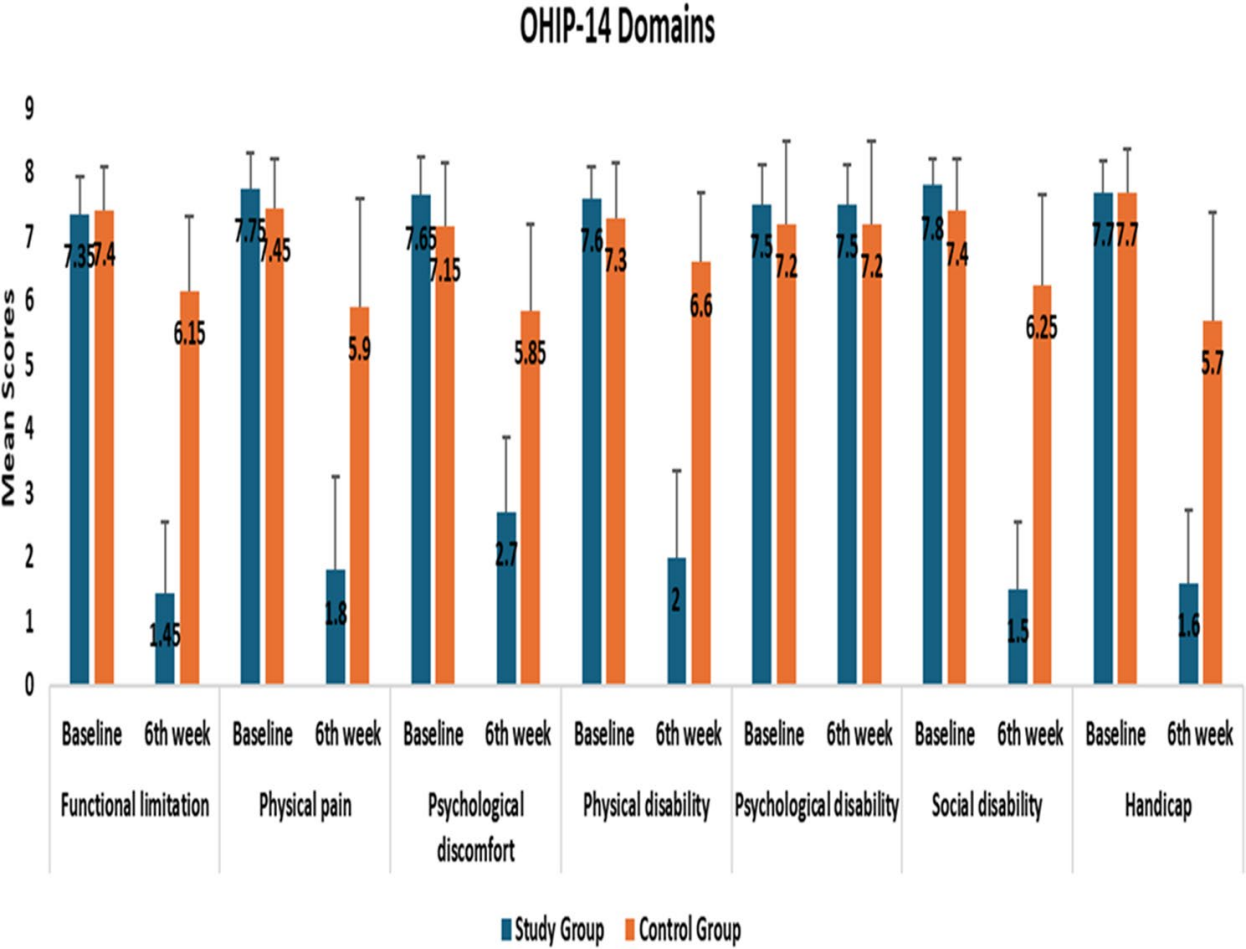
Comparison of OHIP-14 domains at baseline and after 6 weeks between the study groups

OHIP-14 subscale, improved significantly in the study group over six weeks. At baseline, both groups had comparable scores across all domains (*p* > 0.05). By week six, the study group showed significant reductions in all OHIP-14 domains, including functional limitation (from 7.80 ± 1.20 to 2.10 ± 0.56), physical pain (7.85 ± 1.13 to 1.95 ± 0.73), psychological discomfort (7.75 ± 0.88 to 1.95 ± 0.56), physical disability (7.50 ± 1.02 to 1.90 ± 0.83), psychological disability (7.85 ± 0.99 to 1.75 ± 0.62), social disability (7.85 ± 0.88 to 1.85 ± 0.69), and handicap (6.40 ± 0.86 to 1.75 ± 0.54) (all *p* < 0.001). In contrast, the control group showed smaller, nonsignificant changes in these domains. These results demonstrate that NAC nano-spray treatment significantly enhanced multiple aspects of oral health–related quality of life compared to conventional treatment.as shown in supplemental Tables 4 and Fig. 4.

**Fig. 5:**
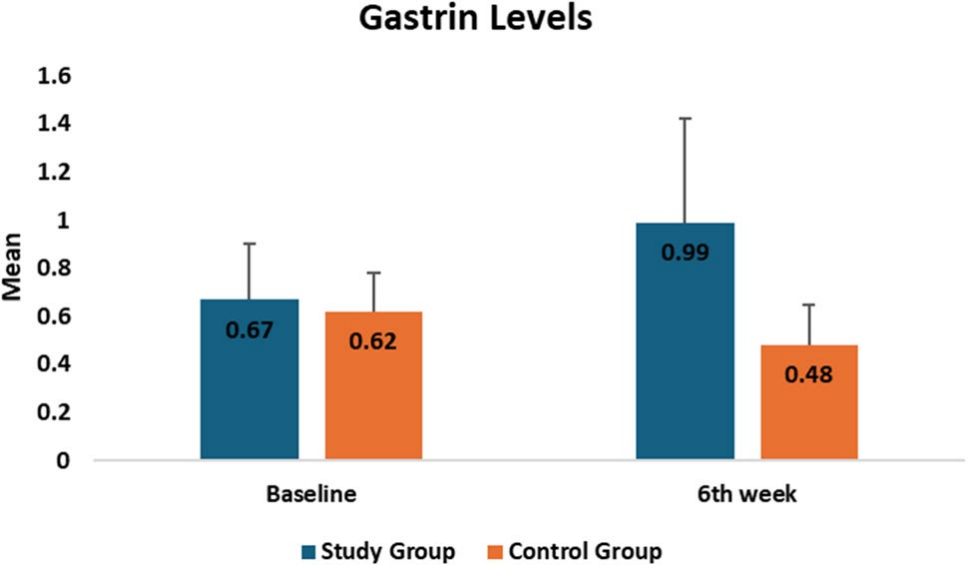
Comparison of serum gastrin levels between the two groups at different timepoints

Serum Gastrin-17 levels were comparable between groups at baseline (study: 0.67 ± 0.23 vs. control: 0.62 ± 0.16; *p* = 0.601). After six weeks, the study group showed a significant increase in Gastrin-17 levels to 0.99 ± 0.43, while levels in the control group decreased to 0.48 ± 0.17, with a significant difference between groups (*p* < 0.001). Both groups exhibited significant within-group changes from baseline (study: *p* < 0.001; control: *p* = 0.004). These results indicate that NAC nano-spray treatment significantly increased serum Gastrin-17 compared to conventional management as presented in Table 6, Supplemental Table 3 and Fig. 5.

**Table 6:** Comparison of serum gastrin levels between study and control groups

Follow up		Study Group (*n* = 20)	Control Group (*n* = 20)	*p* value
Baseline	Mean ± SD	0.67 ± 0.23	0.62 ± 0.16	0.601
	Median (IQR)	0.65 (0.34)	0.61 (0.23)	
6th week	Mean ± SD	0.99 ± 0.43	0.48 ± 0.17	< 0.001*
	Median (IQR)	0.88 (0.67)	0.42 (0.16)	
*p* value^2^		< 0.001*	0.004*	

*Statistically significant difference at p value < 0.05

Table 7 presents the correlation between oral mucositis severity and serum gastrin-17 levels in both the study and control groups. A moderate negative correlation was observed in the study group, indicating that higher gastrin levels were associated with lower mucositis severity. This correlation was statistically significant (*p* < 0.05), with the strength of the relationship measured using Spearman’s rho correlation coefficient.

**Table 7:** Correlation between oral mucositis severity and gastrin levels for the study and control groups

	Study Groups		Control Groups	
	rho	*p* value	rho	*p* value
OMAS	-351	0.027*	0.020	0.901
WHO	-0.301	0.059	0.0120	0.461

*Statistically significant difference at *p* value<0.05, rho: Spearman correlation coefficient

## Discussion

(RIOM) is a debilitating condition marked by erythema, edema, and ulceration of the oral mucosa. It often occurs as a complication of head and neck radiation therapy (RT), chemotherapy, chemoradiotherapy, or hematopoietic stem cell transplantation (HSCT) [44], typically appearing around the third week of treatment and lasting 7 to 98 days [13].

RIOM typically starts as acute inflammation of the oral mucosa, tongue, and pharynx, with the soft palate most severely affected, followed by the hypopharynx, floor of the mouth, buccal mucosa, tongue, and lips [45].

N-acetylcysteine (NAC), a derivative of L-cysteine with mucolytic and antioxidant properties, is therapeutically useful in conditions including RIOM, liver toxicity, respiratory disorders, and certain psychiatric disorders [46]. This study evaluated the efficacy of NAC nano-spray for RIOM and its relationship with serum gastrin-17 (G-17) levels.

The NAC-chitosan nanoparticles exhibited a high positive zeta potential (35.8 mV), indicating good physical stability and preventing particle aggregation. The positive charge also enhances electrostatic interaction with negatively charged mucin on the oral mucosa, supporting improved adhesion and retention [47, 48].

Recent clinical evidence in hematopoietic stem cell transplantation shows that parenteral NAC reduces the incidence and duration of severe oral mucositis. Ongoing trials of NAC formulations, including mouthwash and spray, further support its potential as a supportive therapy in cancer patients [49].

Systematic reviews of topical and supportive therapies for oral mucositis report variable efficacy of interventions, including structured oral care, cryotherapy, and nutraceutical supplementation, in reducing severity or improving quality of life [50].

OHIP assessment showed that NAC nano-spray improved patient-reported physical pain, functional limitation, and psychological discomfort, indicating benefits beyond mucosal healing and positive effects on daily functioning.

Although subjective, OHIP scores provide insight into the real-world impact of NAC treatment, complementing clinical measures of mucositis and supporting its role in symptom relief and overall well-being [51–53].

These results align with Won et al. [29], who reported improved quality of life with NAC inhalation during head and neck radiotherapy, supporting its antioxidant and mucosal protective effects.

The observed therapeutic effects of NAC may result from its antioxidant and anti-inflammatory actions, including ROS scavenging and modulation of pro-inflammatory cytokines [54]. Chitosan in the nanoformulation likely enhanced mucosal adhesion and prolonged tissue contact, further improving efficacy.

Clinical improvement in the NAC group was reflected in WHO oral mucositis scores, which combine subjective symptoms and objective signs for comprehensive severity assessment [55, 56]. Our findings are consistent with Lalla et al. [14], who reported that topical NAC significantly reduced oral mucositis severity during radiotherapy. No contradictory evidence has been published, though studies remain limited.

Serum gastrin-17, secreted by gastric antrum G cells, is influenced by food intake, G-cell number, and gastric pH. It serves as a sensitive marker of antral secretory function and is used in diagnosing gastric cancer and atrophic gastritis [25].

Studies show that improved oral intake and reduced inflammation during mucositis correlate with rising serum G-17 levels. suggesting G-17 as a marker of nutritional status and mucosal recovery, indirectly reflecting NAC’s antioxidant and anti-inflammatory effects [25, 57].

Additional research has shown that COX-1 and COX-2, prostaglandins, and EP receptors are key in gastrointestinal mucosal defense and repair, with COX-1 mediating protection and COX-2 supporting healing. Serum gastrin regulates prostaglandin secretion, underscoring its role in mucosal integrity and therapeutic strategies [58].

Biochemical analysis showed a significant increase in serum G-17 after six weeks in the NAC group, indicating improved mucosal healing and oral intake. A moderate negative correlation with RIOM severity suggests G-17 rises as mucositis resolves.

Although limited, Wu et al. [25] reported that baseline serum G-17 levels correlate with oral mucositis severity, suggesting G-17 as a potential predictor of mucositis risk during radiotherapy.

To the best of our knowledge, this study offers several notable strengths; This study is the first to evaluate NAC nano-spray for RIOM in oral cancer patients, assessing outcomes clinically and via serum G-17. Chitosan-based formulation likely enhanced mucoadhesion, retention, and efficacy, providing comprehensive evidence supporting topical NAC as a promising RIOM therapy.

### Limitations

Despite encouraging results, this study has several limitations. The control group received multiple standard-of-care agents, making it difficult to attribute benefits solely to NAC nano-spray. A placebo-controlled design was not feasible due to ethical concerns. The single-blind design with subjective outcomes may introduce bias, although assessor calibration was implemented. Serum G-17 analysis was exploratory; while associations with NAC were observed, causal or mechanistic conclusions cannot be drawn.

Additionally, the NAC group lacked routine antifungal prophylaxis, unlike the control group, which could have confounded mucositis severity assessments.

### Recommendations for future research

Larger, multicenter studies with longer follow-up and standardized variables (e.g., age, diagnosis, radiation dose) are needed to confirm these findings. Future research should standardize antifungal prophylaxis, explore NAC’s preventive potential in RIOM, and investigate its role in managing other radiation-induced complications, such as xerostomia.

## Conclusions

Topical nano-formulated NAC showed beneficial effects in the management of radiation-induced oral mucositis, reducing mucositis severity, pain, and improving quality of life. The observed changes in serum Gastrin-17 should be interpreted cautiously, considered exploratory and likely to reflect improved mucosal integrity and oral intake rather than a direct effect of NAC. Further studies are warranted to confirm these findings.

## Supplementary information

Supplementary Material 1: Fixed effects from the linear mixed-effects model for oral mucositis. Supplemental table 2. Fixed effects from the linear mixed-effects model for OHIP. Supplemental table 3. Fixed effects from the linear mixed-effects model for gastrin levels. Supplemental table 4. Comparison of OHIP-14 domains at baseline and after 6 weeks between the study groups.

Supplementary Material 2.

## Data Availability

All data included in this study are available from the corresponding author upon request and no third party.

## Abbreviations

RIOM: Radiation-Induced Oral Mucositis
RT: Radiotherapy
NAC: N-acetylcysteine
WHO: World Health Organization
OHIP-14: Oral Health Impact Profile − 14
OM: Oral mucositis
ROS: Reactive Oxygen Species
LLLT: Low Level Laser Therapy
PBMT: Photobiomodulation Therapy
NF-KB: Nuclear factor kappa B
G-17: Gastrin-17
PG-1: Prostaglandin 1
PG-2: Prostaglandin 2
CSNAC: Chitosan-NAC
HPLC: High Performance Liquid Chromatography
DMEM: Dulbecco Modified Eagles Medium
SD: Standard deviation
IQR: Interquartile range
