# Supplementary material for: N-acetylcysteine nano-spray versus conventional treatment in the management of radiotherapy-induced oral mucositis in oral cancer patients: a randomized clinical trial
Source: BMC Oral Health. 2026 Mar 20;26:616. doi: 10.1186/s12903-026-07959-7 (PMC13064302; doi:10.1186/s12903-026-07959-7)
Supplement: Supplementary file 1 — Supplementary Material 1: Fixed effects from the linear mixed-effects model for oral mucositis. Supplemental table 2. Fixed effects from the linear mixed-effects model for OHIP. Supplemental table 3. Fixed effects from the linear mixed-effects model for gastrin levels. Supplemental table 4. Comparison of OHIP-14 domains at baseline and after 6 weeks between the study groups. [file 12903_2026_7959_MOESM1_ESM.docx]

**Supplemental table 1: Fixed effects from the linear mixed-effects model for oral mucositis**

| **Source** | **Numerator df** | **Denominator df** | **F test** | **P value** |
| --- | --- | --- | --- | --- |
| Intercept | 1 | 36.000 | 0.535 | 0.469 |
| Groups | 1 | 36.000 | 0.952 | 0.336 |
| Time | 1 | 38.000 | 57.674 | <0.001* |
| Interaction | 1 | 38.000 | 30.712 | <0.001* |
| Radiation | 1 | 36.000 | 0.021 | 0.885 |
| Age | 1 | 36.000 | 2.241 | 0.143 |

*Statistically significant at p value<0.05

**Supplemental table 2: Fixed effects from the linear mixed-effects model for OHIP**

| **Source** | **Numerator df** | **Denominator df** | **F test** | **P value** |
| --- | --- | --- | --- | --- |
| Intercept | 1 | 53.303 | 20.132 | <0.001* |
| Groups | 1 | 50.809 | 99.073 | <0.001* |
| Time | 4 | 135.419 | 145.440 | <0.001* |
| Interaction | 4 | 135.419 | 58.971 | <0.001* |
| Radiation | 1 | 53.313 | 0.202 | 0.655 |
| Age | 1 | 53.313 | 2.317 | 0.160 |

*Statistically significant at p value<0.05

**Supplemental table 3: Fixed effects from the linear mixed-effects model for gastrin levels**

| **Source** | **Numerator df** | **Denominator df** | **F test** | **P value** |
| --- | --- | --- | --- | --- |
| Intercept | 1 | 36.000 | 0.055 | 0.816 |
| Groups | 1 | 36.000 | 9.586 | 0.004* |
| Time | 1 | 38.000 | 7.160 | 0.011* |
| Interaction | 1 | 38.000 | 48.519 | <0.001* |
| Radiation | 1 | 36.000 | 0.295 | 0.590 |
| Age | 1 | 36.000 | 0.522 | 0.474 |

*Statistically significant at p value<0.05

**Supplemental table 4 Comparison of OHIP-14 domains at baseline and after 6 weeks between the study groups**

*Statistically significant difference at p value<0.05
